# Use of Corticosteroids in the management of Idiopathic Pulmonary Haemosiderosis: Do we have enough evidence

**DOI:** 10.12669/pjms.312.7135

**Published:** 2015

**Authors:** Ammara Mushtaq, Subika Khatoon, Muhammad Asif Qureshi

**Affiliations:** 1 Ammara Mushtaq, Final Year Medical Student, Dow Medical College, Dow University of Health Sciences, Karachi – Pakistan; 2 Subika Khatoon, Final Year Medical Student, Dow Medical College, Dow University of Health Sciences, Karachi – Pakistan; 3 Dr. Muhammad Asif Qureshi, MBBS, PhD Assistant Professor Immunology, Dow Medical College, Dow University of Health Sciences, Karachi – Pakistan

**Keywords:** Idiopathic Pulmonary Haemosiderosis, Corticosteroids

## Abstract

Idiopathic Pulmonary Haemosiderosis (IPH) is a rare disease commonly affecting the paediatric population with approximately 500 globally reported cases in the literature. The disease usually presentswith a symptom triad consisting of ferropenic anaemia, cough with haemoptysis and diffuse bilateral alveolar infiltrates. Therapeutic options for this disorder are not only limited but also not fully effective. Moreover, corticosteroids remain the mainstay of IPH treatment. This communication reviews the available evidence in support of corticosteriod usage in the treatment of IPH. We conclude that the use of corticosteroid in IPH treatment is unfathomed and demands further investigation.

Idiopathic Pulmonary Haemosiderosis (IPH), first described in 1851 by Virchow as ‘’brown lung in duration’’ used to bean incidental finding at autopsy. It is a rare disease with an incidence of about 0.24 and 1.23 cases per million according to a Swedish and a Japanese review, respectively^1^ and approximately 500 cases have been reported in the literature.^1^ The disease occurs mainly in the paediatric age group,^2^ although a number of adult cases have been reported. The aetiology and pathogenesis of the disease is largely unknown to date. IPH manifests itself as a triad comprising of ferropenic anaemia, haemoptysis associated cough and diffuse bilateral alveolar infiltrates. The course of the disease is usually variable, intermittent and progressive, and is characterized by a range of signs/symptoms including attacks of chronic cough, dyspnoea, fatigue, pallor or cyanosis, weight loss, tachycardia, vomiting, haematemesis, acute right-sided heart failure and engorged neck veins. Iron deficiency anaemia, due to the recurrently occurring haemorrhages in pulmonary blood capillaries can, however, be the sole presentation of the disease.^3^ The diagnosis is completed/assured by the identification of haemosiderin-laden macrophages in the lung alveoli, sputum, bronchoalveolar lavage, lung biopsy specimens and gastric washings.^4^ Various therapeutic modalities have been tried for IPH including the use of inhaled and systemic corticosteroids, administration of immunosuppressant drugs and blood transfusions. Nevertheless, the efficacy of these therapeutic options is largely un-explored.

Although a considerable proportion of patients with IPH are prescribe with corticosteroids, the role/efficacy of corticosteroids in IPH remains unclear. Several studies have reported the use of steroids in high doses (e.g., prednisone 2-5 mg/kg/d or equivalent) at initial phase of IPH management.^5^,^6^ In between attacks, corticosteroid maintenance therapy has been used and a number of results validated. A retrospective review of 23 children in whom IPH was diagnosed and steroids in low dose have been tried after the initial high dose on presentation, showed milder form of illness, prolonged survival and reduced exacerbations.^7^ This suggests that patients with IPH show an excellent response to systemic steroids, at least during the haemorrhagic episodes. However, the efficacy of this treatment in the long term steroid therapy has not been explored. In their review, Soergel and Sommers^8^ concluded that despite its usefulness during the acute bleeding episodes, steroid therapy was not of much importance in long term treatment of the disease. Steroids, when given in combination with other immunosuppressive drugs prove to be beneficial as part of the IPH maintenance therapy.^1^ Also, the administration of inhaled corticosteroid, beclomethazone dipropionate, have shown better responses than maintenance therapy on systemic steroids in the case of two children reported by Ban Halima N. and co-workers in their study.^9^ According to a case report, corticosteroid therapy has also been effective in preventing the episodes of haemoptysis in a patient who was initially maintained on a 20mg dose of prednisolone that was then tapered to 10mg dose of the drug one month later.^10^ In a case of IPH from Karachi, Pakistan^11^ coritcosteroids proved to be effective in an 8 year old child who presented with severe iron deficiency anaemia. Another success of corticosteroid treatment was reported in a case from India where the patient showed remarkable recovery within 10 days of therapy.^12^

In contrast to the aforementioned studies, a study documented that the administration of corticosteroids to a 19-year old male worsened the respiratory function and he experiences IPH remission within 3 years of lung transplantation.^13^ Similarly, there are studies that make a cogent case that prolonged survival of the patients was dependent on long term immunosuppressant drugs along with steroids, and corticosteroids alone were not sufficient for the long term treatment of the disease^14^ and their side-effects in long term therapy cannot be ignored.^8^ Also, old age (compared to the typical age of 1-7 years) at diagnosis has been associated with poor response to the therapy,^4^ making corticosteroid administration questionable in such cases. Other investigational treatment options include immunosuppressive agents like azathioprine, hydroxycholoroquine, cyclophosphamide and methotrexate, with variable success rates.^1^ Four patients in a study were prescribed azathioprine when they didn’t respond to prednisolone.^4^

In summary, this manuscript highlights the facts that the use of corticosteroids in IPH management is a heavily debated topic that needs further investigation to clarify the existing conflicts. In our opinion, this issue can be resolved either by using alternate treatment options including immuno suppressive agents like azathioprine, hydroxycholoroquine, cyclophosphamide, methotrexate as well as azathioprine, which has been used in patients who were non responsive to prednisolon^1^ or running clinical trials to prove or disprove the efficacy of corticosteroid treatment. However, it should be noted that due to rarity of the disease, it has been and will be difficult to conduct prospective clinical trials.^1^ Therefore, much of the understanding on this considerably disputed issue will rely on case reports explaining the course of the disease and the therapeutic response. Subsequent meta-amylases of these case reports will further augment the understanding of this debated issue. Moreover, we emphasize upon active research in order to investigate the cause of this disorder so that selective, and directed, therapeutic approaches can be undertaken.
